# Autistic Children/Adolescents Have Lower Adherence to the Mediterranean Diet and Higher Salivary IL-6 Concentration: Potential Diet–Inflammation Links?

**DOI:** 10.3390/pathophysiology31030028

**Published:** 2024-07-28

**Authors:** Milagros Fuentes-Albero, Mayra Alejandra Mafla-España, José Martínez-Raga, Omar Cauli

**Affiliations:** 1 Clinica Ripalda, 46001 Valencia, Spain; milafuentesalbero@gmail.com; 2 Nursing Department, University of Valencia, 46010 Valencia, Spain; maymaes@alumni.uv.es; 3 Department of Psychiatry and Clinical Psychology, Hospital Universitario Doctor Peset and University of Valencia, 46010 Valencia, Spain; martinezragaj@gmail.com

**Keywords:** diet, inflammation, neurodevelopmental disorders, cytokine, biomarker stress, children

## Abstract

Background: Autism spectrum disorder (ASD) is one of the most prevalent neurodevelopmental disorders. Many patients with ASD often show behavioral problems at mealtimes, including food selectivity and atypical feeding behaviors. The Mediterranean diet (MD) has a beneficial effect on mental health for the general population across different ages. There is evidence that good adherence to the MD is effective in reducing peripheral inflammatory markers, such as the cytokine interleukin-6 (IL-6). The present study was designed to evaluate adherence to the MD in children with ASD using age- and sex-matched, typically developing individuals (TDs) as a control group and to determine whether differences in adherence to the MD are associated with salivary IL-6 and IL-6 receptor concentration. Methods: Twenty children and adolescents with ASD (mean age 9.95 ± 0.65 years) and twenty TDs (mean age: 9.85 ± 0.59 years) participated in this study (N = 16 males and N = 4 females in each group). Participants with ASD were enrolled in a psychiatric consultation in Valencia (Spain), and TDs were recruited from two public schools in Valencia. The parents of both ASD and TD groups answered the items in a validated Mediterranean Diet Quality Index for children and adolescents (KIDMED) questionnaire on their children’s adherence to the MD. Results: The mean adherence to MD score was significantly lower in the ASD group (9.10 ± 0.42) (range 6–12) than in the TD group (10.35 ± 0.31) (range 8–12) (*p* = 0.02, Mann–Whitney U test). There was no statistically significant association between adherence to the MD and age or sex in both groups, but there was a significant correlation between the total KIDMED score and body mass index (BMI) in the ASD group. Regarding the concentration of Il-6 and the Il-6 receptor in saliva samples, there were no significant differences between the two groups; however, linear regression analysis by group revealed significant associations between the adherence to MD score and the concentration of IL-6 and its receptor in saliva in the ASD group (*p* = 0.003, OR = 0.68, 95% CI 0.007 to −0.02; *p* = 0.009, OR = −0.64, 95% CI −0.01 to −0.00). In contrast, no significant associations were observed between the adherence to MD score and the concentration of IL-6 and its receptor in saliva in the TD group. Conclusions: Children and adolescents with ASD showed significantly lower adherence to the MD, which can contribute to nutritional deficits described in ASD, and the role of BMI composition (fat versus lean mass) needs to be further investigated in this group. The concentration of IL-6 and its receptor in saliva is associated with adherence to the MD, suggesting a possible link between IL-6 and diet in ASD. Further studies to clarify the associations between IL-6, psychiatric alterations, and diet in ASD are needed.

## 1. Introduction

Autism spectrum disorder (ASD) is a common neurodevelopmental condition characterized by the core symptoms of poor social–emotional reciprocity and repetitive, stereotyped behaviors [1]. Its prevalence has increased significantly in the last few decades [2,3]. In 2010, the overall prevalence of ASD in the United States was 14.7 cases per 1000 (1 in 68) children 8 years of age, and the male-to-female ratio was 5:1 [4]. An analysis of the literature on ASD prevalence studies published since 2014 confirms an increase in the prevalence, but there is high variability in the estimates of prevalence worldwide due to methodological differences among the studies on how cases are detected, which population is studied, and, to a lesser extent, how cases are defined [4]. The results of the European Union’s Autism Spectrum Disorder in Europe project (ASDEU—https://www.autismeurope.org/programme-asdeu-autism-spectrum-disorders-in-the-european-union-2015-2017/ accessed on 15 May 2024), an ad hoc study performed in Italy, yielded an estimated prevalence of 0.80%, using only the number of children certified with ASD or with other neurodevelopmental disorders in comorbidity with ASD. This prevalence rose to 1.04% when children identified through the screening procedure were included and to 1.15% based on a probabilistic calculation to adjust for non-responses [5]. A recently published study conducted in northern Spain within the ASDEU project yielded an estimated prevalence of 0.58% [6].

Eating is among the most essential of human activities, and it is necessary not only to sustain life but also to ensure proper development. Indeed, our diet is the main source of nutrients for the body. For the brain in particular, nutrients such as omega-3 fatty acids, docosahexaenoic acid, iron, iodine, calcium, folic acid, and vitamin D [7,8,9] are important, especially during the prenatal and perinatal periods.

In addition to the core symptoms, ASD may have strong associations with other disorders and/or be associated with a plethora of behaviors and symptoms, such as those related to food selectivity and consequent inadequate dietary intake [10,11].

Although some types of eating disturbances, such as food refusal, are also frequent in the general pediatric population, their prevalence appears to be significantly higher in children with ASD, with rates ranging from 51% to 89% [12].

Children with ASD often show behavioral problems at mealtimes, including food selectivity, as has been reported by many studies over the years. However, “food selectivity” is used in the literature to refer to a wide range of dysfunctional nutritional habits and behaviors, including food refusal, a limited repertoire of accepted foods, single-food intake, excessive intake of a few foods [10,13], and selective intake of certain food categories (such as carbohydrates, fats, or proteins) [14]. Atypical feeding behaviors, the intentional adoption of diet restrictions, and the lifestyles of individuals with ASD (not only including different levels of physical activity but also idiosyncratic social skills and poor social interaction) are factors that imply risks of either excessive or insufficient nutrient intake [15,16]. In addition, the nutritional status of individuals with ASD can influence the presentation of symptoms, especially in cases with deficient dietary intake [17]. Moreover, increased central and peripheral inflammation and oxidative stress have been linked to the pathophysiology of ASD [18,19]. The interest in research on immune system alterations in ASD, including aberrations in cytokine profiles and signaling, has increased recently [20,21,22,23]. The inflammatory state associated with ASD has often been linked to immune system dysfunction [24]. Enhanced inflammatory activity in children with ASD has been demonstrated through pro-inflammatory biomarker analysis. Interleukins are signaling proteins belonging to the cytokine family, responsible for immune modulation and inflammatory responses [25]. Pro-inflammatory cytokines, such as IL-1β, IL-6, and IL-8, in the blood were found to be elevated in the plasma of children with ASD compared to the age-matched TD control group or to children with other developmental disabilities [26,27,28]. Increased pro-inflammatory cytokine levels have been associated with impairments in stereotypical behaviors and in regression, suggesting that dysfunctional immune responses could affect core behaviors in ASD [26].

The Mediterranean diet (MD) is a largely plant-based diet containing large amounts of fruits, vegetables, whole grains, olive oil, nuts, and seeds as rich sources of antioxidants, fibers, phytochemicals, and omega-3 fatty acids [29,30]. The MD is considered useful in preventing and treating cardiovascular diseases and inflammatory disorders [31]. Previous studies have demonstrated that high levels of consumption of phytochemical-rich foods and omega-3 can improve symptoms in other neurodevelopmental disorders, such as attention deficit hyperactivity disorder (ADHD) [32,33].

The MD has been associated with an improvement in inflammatory markers and oxidative stress compared to diets high in carbohydrates and saturated fats [34,35]. It is therefore considered to have anti-inflammatory and antioxidant properties [36]. Some of the advantages demonstrated by the MD include reduced oxidative stress, increased insulin sensitivity, lower LDL cholesterol levels, increased genomic stability, improved immune system function, improved production of metabolites by the gut microbiota, and reduced inflammation [37]. This diet has also been shown to improve mitochondrial function and reduce cellular oxidative stress [38]. The Mediterranean diet also decreases the plasma concentrations of C-reactive protein, as well as of cytokines, such as the inflammatory interleukins (ILs) IL-6, IL-7, IL-18, and TNF-α (tumor necrosis factor alpha) [35,39].

Only two papers have evaluated the MD in children with ASD [40,41]. In a matched case–control study performed in Spain, it was found that children with ASD had lower saturated fatty acid and omega-3 polyunsaturated fatty acid intake compared to typically developing children [40]. Children with ASD also failed to meet dietary recommendations for thiamin, riboflavin, vitamin C, or calcium [41].

In this paper, we evaluated adherence to the MD in children/adolescents with ASD to assess whether the level of adherence is associated with changes in the peripheral concentration of the pro-inflammatory cytokine IL-6 and its receptor. We focus on interleukin-6 because several studies have reported higher IL-6 levels in preclinical models of ASD and in patients with ASD compared to the general population [42,43] and because changes in these cytokines have been reported with adherence to the MD in the general population [44,45].

## 2. Methods

### 2.1. Participants

The present observational case–control study was conducted in a sample of children and adolescents with ASD attending child psychiatry outpatient care in Valencia (Spain) during 2021–2022. Neurologically healthy sex- and age-matched children and adolescents (control group) were likewise recruited from two public schools in Valencia (Spain). Autism spectrum disorder was confirmed by an expert psychiatrist based on *Diagnostic and Statistical Manual of Mental Disorders* (DSM-5) diagnostic criteria. The parents of children with ASD were interviewed during ordinary appointments with the child psychiatrist. Clinical information (diagnosis of ASD, medication, comorbid disorders, anthropometric data) was retrieved by reviewing the medical records in the psychiatrist’s surgery.

Body mass index (BMI) was calculated as weight in kilograms divided by the square of height in meters. BMI is age- and sex-specific for children and adolescents and is often referred to as BMI-for-age. Following international guidelines, the BMIs were grouped into four categories: underweight (BMI below the 5th percentile), healthy weight (BMI at or above the 5th percentile and less than the 85th percentile), overweight (BMI equal to or greater than the 85th percentile but less than the 95th percentile), or obese (BMI at or above the 95th percentile) [46,47,48]. The children and adolescents in the control group were sex- and age-matched (proportion 1:1) to the children and adolescents in the ASD group. Matching increases the efficiency of the estimates if the matching variables are associated with both the disease and diet habits [49].

The study protocol followed the rules of the Declaration of Helsinki and was approved by the Ethics Committee of the University of Valencia (Valencia, Spain) (protocol number H1397475950160). Data were obtained in face-to-face interviews between the researcher and interviewees (parents), and the research aims were explained in order to facilitate voluntary informed consent. The parents signed the written informed consent form before participating in this study.

### 2.2. Mediterranean Diet Adherence Questionnaire

The Spanish version of the Mediterranean Diet Quality Index for children and adolescents (KIDMED questionnaire) [50] was used to evaluate adherence to the MD. This self-reported instrument consists of 16 questions designed to evaluate eating habits. Each question offers two response options, “yes” or “no”, with a rating on a scale ranging from −1 (negative connotation) to +1 (positive connotation). Twelve of the sixteen questions are scored positively, and four are scored negatively. The total KIDMED scores range between 0 and 12, classified as follows: ≥8 points indicate good adherence (optimal MD); 4–7 points indicate average adherence; and ≤3 points indicate poor adhesion.

### 2.3. Measurement of Salivary IL-6 and IL-6 Receptor

Saliva samples were collected using the Salivette^®^ device (Sarstedt, 51588 Nümbrecht, Germany) to assess the levels of IL-6 and its receptor. The participants’ parents were asked to ensure that the participants refrained from eating, drinking, or performing oral hygiene procedures for at least one hour before saliva collection. Each sample was centrifuged to remove mucins, insoluble material, and cellular debris. The resulting supernatant was aliquoted into Eppendorf tubes and frozen at −80 °C for further analysis. ELISA immunoassays were performed using the high-sensitivity human ELISA kit for IL-6 and its receptor (reference number Ab178013 and Ab46029, respectively), following the instructions of the manufacturer, using 100 µL samples brought to room temperature.

### 2.4. Statistical Analysis

The sample size of the study population was calculated and estimated using two series of model correlation tests with G*Power 3.1.9.2 software (G*Power©; Dusseldorf University, Düsseldorf, Germany). A moderate correlation coefficient of r > 0.30 [51,52], a two-tailed hypothesis, an error of α = 0.05 with a confidence interval of 95% and β error = 20%, and power analysis of 1 − β = 0.80 were also considered. A sample size of 40 individuals was therefore considered appropriate for this study. Frequency distribution was explored for the qualitative variables, while for the quantitative variables, measures such as the arithmetic mean, the standard error of the mean (SEM), and the range of values were calculated. The normality of each variable was evaluated using the Shapiro–Wilk test. Since no variable showed a normal distribution, non-parametric statistical methods were used. The categorical variables were compared between groups using the chi-squared test. The Mann–Whitney U test was used to compare the differences between independent groups in the case of ordinal or continuous dependent variables. The correlations between continuous variables were investigated using Spearman’s correlation coefficient, and Pearson’s chi-square test was used to evaluate the statistical relationship between two categorical variables. A linear regression analysis was also performed to explore significant associations with certain independent variables. Version 26.0 of the SPSS statistical package (SPSS, Inc., Chicago, IL, USA) was used throughout. Statistical significance was considered when *p* < 0.05.

## 3. Results

### 3.1. Sample Characteristics

Forty children and adolescents with autism spectrum disorder (ASD) and a sex- and age-matched control group (TD) were evaluated in this study. Age, sex distribution and BMI are shown in Table 1. No significant differences were observed in BMI as a continuous variable between the ASD and TD groups (*p* = 0.127, Mann–Whitney test), nor as a categorized variable (*p* = 0.607, Chi-square test). During the study, 12 patients with ASD (60%) were receiving psychotropic medication, while the remaining 8 (40%) were not receiving any medication. None of the young people in the TD group were receiving any psychotropic medication. In the ASD group, 4 (20%) were in primary school, 6 (30%) were attending secondary school, and 10 (50%) were attending a special education school.

### 3.2. Comparison of Adherence to the MD between the ASD and TD Groups

**Figure 1:**
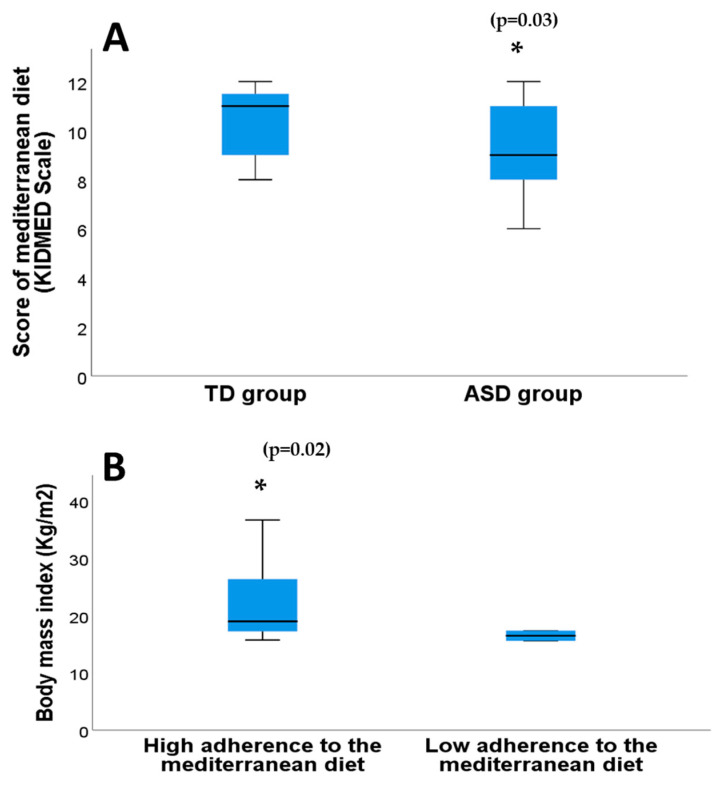
Adherence to the MD in the ASD and TD groups (**A**) and adherence to the MD in the ASD group based on body mass index (**B**). * Significant differences (*p*-value in parentheses).

The mean adherence to MD score was significantly lower in the ASD group (9.10 ± 0.42) (range 6–12) than in the TD group (10.35 ± 0.31) (range 8–12) (*p* = 0.02, Mann–Whitney U test) (Figure 1A) based on the cut-off score of ≥8 points indicating good adherence on the KIDMED scale. Furthermore, there were significant differences in high-adherence rates between the two groups (*p* = 0.03, Pearson’s chi-square test); it was 80% among the participants in the ASD group, while all the participants in the control group showed high adherence (100%). No significant correlations were found between the total KIDMED score and age in the total group or in the ASD and TD groups individually (Rho = 0.09, *p* = 0.55; Rho = 0.04, *p* = 0.85; Rho = 0.11, *p* = 0.63, respectively, Spearman’s correlation coefficient in all cases).

Likewise, there were no significant differences in the total KIDMED score according to sex within the total sample (*p* = 0.43) nor between the two study groups (*p* = 0.38 for the ASD group versus *p* = 0.75 for the TD group) (Mann–Whitney U test in all cases). There was no statistically significant correlation between the total KIDMED score and BMI in the total sample (*p* = 0.22, Mann–Whitney U test), though the correlation between the total KIDMED score and BMI in the ASD group was significant (*p* = 0.02, Mann–Whitney U test).

### 3.3. Comparison of Salivary IL-6 and IL-6 Receptor Concentration between Groups and Sociodemographic and Clinical Variables

The mean concentration of IL-6 in saliva for the whole sample was 88.6 ± 11 pg/mL (SEM) (range 11.8–243), and the mean IL-6 receptor concentration was 204.9 ± 19.2 pg/mL (SEM) (range 72.09–559). No significant differences were found in the salivary concentration of IL-6 and IL-6 receptor between the two groups (*p* = 0.25 and *p* = 0.77, respectively, Mann–Whitney U test).

**Figure 2:**
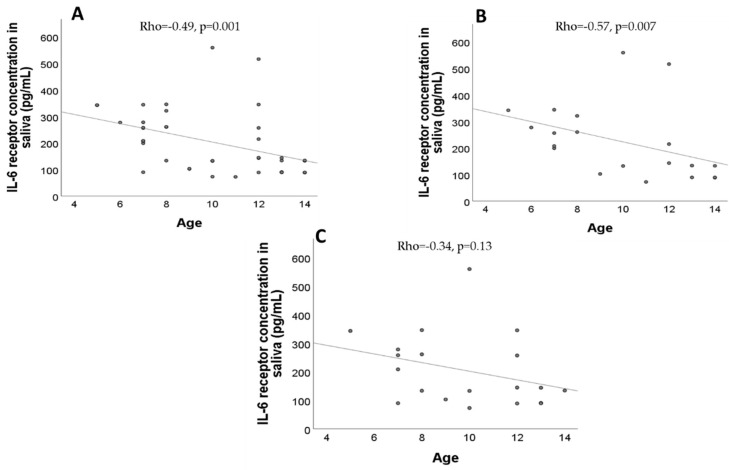
Association between salivary IL-6 concentration and age. (**A**) IL-6 receptor concentration in saliva in the total sample. (**B**) IL-6 receptor concentration in saliva in the ASD group. (**C**) IL-6 receptor concentration in saliva in the TD group.

There were no significant correlations between IL-6 concentration and age in the total sample (Rho = 0.26, *p* = 0.09) nor in the ASD group (Rho = −0.30, *p* = 0.19) and TD group (Rho = −0.25, *p* = 0.27) (Spearman’s correlation coefficient in all cases). However, there were significant correlations between IL-6 receptor concentration and age in the total sample (Rho = −0.49, *p* = 0.001) and in the ASD group (Rho = −0.57, *p* = 0.007). No significant correlations were found between IL-6 receptor concentration and age in the TD group (Rho = −0.34, *p* = 0.13), as seen in Figure 2.

No significant differences were found in the concentration of IL-6 according to sex in the total sample (*p* = 0.24) nor in the ASD group (*p* = 1.00) and TD group (*p* = 0.12) (Mann–Whitney U test in all cases). Similarly, there were no significant differences in IL-6 receptor concentration according to sex in the total sample (*p* = 0.06) nor in the ASD group (*p* = 0.21) and TD group (*p* = 0.17).

Likewise, there were no significant differences in the concentration of IL-6 according to BMI in the total sample (*p* = 0.08) nor in the ASD group (*p* = 0.09) and TD group (*p* = 0.25). Similarly, there were no significant differences in IL-6 receptor concentration according to BMI in the total sample (*p* = 0.06) nor in the ASD group (*p* = 0.32) and TD group (*p* = 0.09) (Kruskal–Wallis test).

### 3.4. Multivariate Analyses

Linear regression analysis was performed to identify significant relationships in a multivariate analysis using clinical and sociodemographic variables. The dependent variable in this analysis was the total Mediterranean diet adherence score according to the KIDMED scale. The independent, or predictor, variables were age, sex, study groups, BMI, and the concentrations of IL-6 and the IL-6 receptor. Significant associations were identified between adherence to the MD within the study groups (*p* = 0.002, odds ratio [OR] = −0.49, 95% CI −2.78 to −0.65) and the concentration of IL-6 (*p* = 0.02, OR = 0.36, 95% CI 0.001 to 0.017). The ASD group also presented lower adherence to the MD and higher IL-6 levels than the TD (control) group. However, no significant associations with the other variables in the total group were observed, as seen in Table 2.

Linear regression analysis of separate groups revealed significant associations between the Mediterranean diet adherence score and the concentration of IL-6 and its receptor in saliva in the ASD group (*p* = 0.003, OR = 0.68, 95% CI 0.007 to 0.02; *p* = 0.009, OR = −0.64, 95% CI −0.01 to 0.00). In contrast, no significant associations were observed with the other variables in this group. In the TD group, no significant associations were found between adherence to the MD and the variables age, sex, BMI, and concentration of IL-6 and its receptor in saliva, as seen in Table 2.

## 4. Discussion

This study explored the impact of the Mediterranean diet on salivary IL-6 levels in individuals with ASD and is a novel study in terms of analyzing the role of the MD in relation to salivary IL-6 levels in individuals with ASD.

Reducing the levels of this cytokine through diet could be relevant in reducing inflammation associated with ASD. Several studies have shown that individuals with ASD have high levels of cellular oxidative stress [53], as well as a dysfunctional immune response mediated by microglia in the central nervous system [54]. A comprehensive meta-analysis showed significant elevation in the peripheral blood levels of the proinflammatory cytokines IL-6, IL-1β, IL-7, and IL-12p70 in patients with ASD versus the controls [55]. Chronic inflammation in the brain during neurogenesis (probably mediated by maternal immune activation during pregnancy) can have a negative impact on brain development, affecting synaptic connectivity, brain plasticity, and neuronal development, which are factors that can condition the emergence of autism spectrum disorder. The elevation of IL-6 stimulates the formation of excitatory synapses while negatively affecting the development of inhibitory synapses [56]. High levels of IL-6 are associated with more severe autism spectrum disorder, a greater presence of stereotyped behavior, greater hyperactivity, and higher levels of intellectual disability [57].

Patients with ASD show an increase in the levels of IL-6 in plasma, peripheral blood cells, and lymphoblasts [42,43]. In the brain, IL-6 has been found to be elevated in the anterior cingulate gyrus and in the cerebrospinal fluid of individuals with ASD [58]. TNF-α, IL-6, and IL-17 levels are also elevated, and IL-2 levels are reduced [54]. In our study, the group of patients with ASD had higher salivary IL-6 levels than the control group, which has not been reported before for this biological fluid.

The effect observed in IL-6 in ASD seems to be associated with adherence to the MD in general, as other studies assessing the association of adherence to the MD with diverse inflammatory biomarkers in healthy persons reported inverse correlations. Higher adherence to the Mediterranean diet was associated with lower levels of inflammatory cytokines in blood, such as IL-6 [44,45,59,60], interleukin-1β [59], IL-18 [60], tumor necrosis factor alpha (TNF-α) [61], and the well-known inflammatory marker, high-sensitivity C-reactive protein [44,61].

Diet can influence the overall well-being of individuals with ASD, who are at risk of suffering from malnutrition due to inadequate intake (selective or restrictive eating), poor nutrient absorption due to intestinal dysbiosis, and intestinal rhythm alterations. These subjects often have low levels of multiple nutrients (pantothenic acid, folate, biotin, vitamin B12, vitamin D, vitamin E, calcium, iron, magnesium, iodine, chromium, copper, zinc, and selenium) compared to the control population [62]. Regarding the lipid profile, individuals with ASD have lower plasma concentrations of omega-3 fatty acids and higher concentrations of omega-6 fatty acids compared to the control population, suggesting a potentially more proinflammatory state [63]. In a study performed in Spain, children with ASD displayed unmet dietary recommendations for omega-3 polyunsaturated fatty acids, thiamin, riboflavin, vitamin C, and calcium intake [40,41].

In addition to having proven physical (improved cardiovascular health) and mental benefits (reduced risk of depression, Alzheimer’s disease, etc.), the Mediterranean diet has been related to an improvement in cognitive function and inflammatory markers in ASD [64]. This diet has been shown to improve mitochondrial function and reduce cellular oxidative stress [38]. It also reduces the plasma concentrations of C-reactive protein and of inflammatory cytokines, such as IL-6, IL-7, IL-18, and TNF-α [35,39].

In the present study, adherence to the MD was lower in children and adolescents with ASD than in the control group. A limitation of our study is that the causes of lower adherence to this type of diet were not analyzed. The data from this study also show that individuals in the ASD group who consumed a Mediterranean diet (better MD adherence) had lower salivary concentrations of IL-6. In the ASD group, those with high adherence to the MD had higher BMIs than those with poor adherence. Since the mean BMI was within the normal value in both groups, and because no significant differences were observed between the ASD and TD groups in terms of children/adolescents with overweight/obesity or with underweight, we speculate that these data may be related to the lean mass amount in the children with ASD who demonstrated higher adherence to the MD. 

A recent study of Spanish university students found that the participants classified as having high adherence to the Mediterranean dietary pattern showed higher BMIs, total lean mass, and muscular strength than the participants with medium and low adherence [65]. Future studies of the AD group need to evaluate lean mass and the amount of physical exercise in order to clarify the association between higher adherence to the MD and BMI in ASD.

In the present study, we decided to ascertain the concentration of Il-6 and its receptors in saliva samples. In most studies of biomarkers related to the etiopathogenesis of autism, markers have been evaluated in blood or plasma, which involves the use of invasive techniques. Given the difficulties in social understanding among individuals with ASD, blood extractions are usually stressful and potentially traumatic. The search for these biomarkers in other body fluids, such as saliva, may therefore be a gentler alternative for these patients. The concentration of biomarkers in saliva may be up to a thousand times lower than in blood [66], although saliva allows for multiple collections without causing pain or creating stress for the individual with ASD [67], thereby facilitating the monitoring of biomarkers in non-cooperative patients [68].

A growing body of evidence suggests that the protective effects of the MD may result, at least in part, from its anti-inflammatory properties [69]. Low-grade chronic inflammation has not only been considered a potential mediator for the development of cardio-metabolic diseases, but it can also influence psychiatric disorders [70]. Interestingly, good adherence to the MD, even in the absence of weight loss, significantly reduces peripheral inflammatory markers [60]. Therefore, studying the relation between diet quality (MD adherence is a gold standard in Mediterranean countries) and inflammatory marker concentration will be interesting to evaluate any effects of psychiatric alterations in individuals with ASD.

The present study presents some limitations that should be taken into account. Limitations of this study include its sample size and the fact that the reasons explaining the difficulties in adherence to the Mediterranean diet in individuals with ASD were not recorded. Despite the small sample size, the results obtained are statistically significant, showing that both IL-6 and its receptor are increased in saliva in the ASD population with lower MD adherence. Future studies comparing a group of children and adolescents without neuropsychiatric disorders but with low MD adherence would help to clarify whether the increase in salivary IL-6 concentration is due to low MD adherence, autism, or both. The influence of the parents’ adherence to the MD in terms of its contribution to their children’s adherence to the MD in both groups was not evaluated, and this clearly needs to be examined in future studies, as well as the effects of various types of psychotropic medications in terms of their influence on IL-6 levels.

## 5. Conclusions

This study offers promising results regarding the potential to improve inflammatory markers in individuals with ASD through dietary interventions. The data suggest that implementing a Mediterranean diet in individuals with ASD may provide the nutrients necessary for enhancing overall cognitive function and reduce inflammation and cellular oxidation processes by lowering the levels of IL-6.

This study is also innovative in terms of its use of salivary markers. The authors believe that this method could be very useful in furthering our understanding of the etiopathogenesis of autism. Studies involving larger samples will be necessary to confirm these initial promising findings, which are aimed at improving the diet and the quality of life of individuals with ASD.

## Figures and Tables

**Table 1:** Sociodemographic and clinical characteristics.

Variables	Frequency (%) Mean + SEM (Range Max–Min) ASD Group: 20	Frequency (%) Mean + SEM (Range Max–Min) TD Group: 20
Age	9.95 ± 0.65	9.85 ± 0.59
Sex:		
Female	4 (20%)	4 (20%)
Male	16 (80%)	16 (80%)
BMI (as continuous variable)	20.9 ± 1.44 (15.5–36.6)	18.2 ± 0.94 (14–27)
BMI (as categorized variable)		
Underweight	2 (10%)	5 (25%)
Normal weight	7 (35%)	7 (35%)
Overweight	6 (30%)	4 (20%)
Obese	5 (25%)	4 (20%)

**Table 2:** Association between adherence to the MD and clinical and sociodemographic variables.

Variable	Group	Standard B Coefficient	t	*p*-Value	95% Confidence Interval Lower Limit–Upper Limit
Age	Total group	0.13	0.80	0.42	−0.13–0.30
Sex		−0.20	−1.19	0.24	−2.36–0.61
BMI		0.30	1.87	0.07	−0.008–0.19
Group		−0.49	−3.29	0.002	−2.78–−0.65
IL-6 concentration in saliva		0.36	2.34	0.02	0.001–0.01
IL-6 receptor concentration in saliva		−0.27	−1.66	0.10	−0.009–0.00
Age	ASD group	0.06	0.32	0.74	−0.22–0.30
Sex		−0.34	−1.66	0.11	−3.64–0.46
BMI		0.30	1.53	0.14	−0.03–0.21
IL-6 concentration in saliva		0.68	3.58	0.003	0.007–0.02
IL-6 receptor concentration in saliva		−0.64	−3.03	0.009	−0.01–−0.00
Age	TD group	0.25	0.76	0.45	−0.23–0.50
Sex		−0.29	−0.87	0.39	−3.46–1.46
BMI		0.20	0.74	0.46	−0.12–0.25
IL-6 concentration in saliva		−0.14	−0.51	0.61	−0.017–0.01
IL-6 receptor concentration in saliva		−0.11	−0.40	0.69	−0.008–0.00

## Data Availability

The data presented in this study are available on request with a scientific purpose from the corresponding author.
